# Guarding against malicious biased threats (GAMBiT) datasets: Revealing cognitive bias in human-subjects red-team cyber range operations

**DOI:** 10.1016/j.dib.2026.112476

**Published:** 2026-01-18

**Authors:** Brandon Beltz, Jim Doty, Yvonne Fonken, Nikolos Gurney, Brett Israelsen, Nathan Lau, Stacy Marsella, Rachelle Thomas, Stoney Trent, Peggy Wu, Ya-Ting Yang, Quanyan Zhu

**Affiliations:** a Bulls Run Group, 9207 Bulls Run Parkway, Bethesda, MD 20817-2403, USA; b Raytheon Technologies, 1000 Wilson Blvd., Arlington, VA 22209, USA; c Institute for Creative Technologies, University of Southern California, 12015 East Waterfront Drive, Playa Vista, CA 90094, USA; d Department of Electrical and Computer Engineering, New York University, New York, NY 10012, USA; e Khoury College of Computer Sciences, Northeastern University, Boston, MA 02115, USA; f Grado Department of Industrial and Systems Engineering, Virginia Tech, Blacksburg, VA 24061, USA

**Keywords:** Cybersecurity dataset, Red team, Cognitive bias, Cyber range, Host and network telemetry, Attacker behavior modeling

## Abstract

We present datasets from three large-scale human-subject experiments involving red-team hacking in a cyber range in the Guarding Against Malicious Biased Threats (GAMBiT) project. Across Experiments 1-3 (July 2024-March 2025), 19-20 skilled attackers per experiment conducted two 8-hour days of self-paced operations in a simulated enterprise network (SimSpace Cyber Force Platform) while collecting multi-modal data: self-reports (background, demographics, psychometrics), operational notes, terminal histories, key logs, network packet captures (PCAP), and NIDS alerts (Suricata). Each participant began from a standardized Kali Linux VM and pursued realistic objectives (e.g., target discovery and data exfiltration) under controlled constraints. Derivative curated logs and labels are included. The combined data release supports research on attacker behavior modeling, bias-aware analytics, and method benchmarking. Data are available via IEEE DataPort entries for Experiments 1-3.

Specifications Table

**Table:** 

Subject	Computer Sciences
Specific subject area	Computer Networks and Communications; Cybersecurity; Human-Subjects; Cyber Operations; Attacker Behavior Modeling; Intrusion Tactics; Cognitive Biases in Cyber Operations; Network and Host Activity Data.
Type of data	Table for questionnaires (CSV/JSON); PCAP files; NIDS alerts (Suricata); host keylogs; Bash/Zsh histories; CherryTree operator notes; curated/clean logs.
Data collection	Live cyber range exercises on the SimSpace Cyber Force Platform with a standardized Kali Linux start box; hourly and end-of-day surveys; host and network sensors (keylogger, shell history, PCAP, Suricata).
Data source location	Data was collected at Blacksburg, VA; stored at NYU
Data accessibility	Repository name: IEEE DataPort Data identification number: Experiment 1 [1]: 10.21227/dwkg-n940 Experiment 2 [2]: 10.21227/39z8-w554 Experiment 3 [3]: 10.21227/xdw9-3677 Direct URL to data: Experiment 1: https://ieee-dataport.org/documents/guarding-against-malicious-biased-threats-gambit-experiment-1 Experiment 2: https://ieee-dataport.org/documents/guarding-against-malicious-biased-threats-gambit-experiment-2 Experiment 3: https://ieee-dataport.org/documents/guarding-against-malicious-biased-threats-gambit-experiment-3 Note that large PCAP files are available only for Experiment 1; PCAP files for other experiments can be provided upon request (email: qz494@nyu.edu).
Related research article	None.

## 1 Value of the Data

• **Multi-modal HSR red-team telemetry**: The dataset provides a comprehensive capture of human-subject red-team (HSR) exercises in a realistic enterprise cyber range. It synchronizes host-level telemetry (e.g., keystroke logs, shell histories, and operating system event traces), network-level data (full packet captures, intrusion detection system alerts, and NetFlow summaries), and human-factors data (psychometric assessments, targeted surveys, operational notes, and self-reports) collected under standardized, repeatable scenarios. This multi-modal alignment enables fine-grained correlation of attacker actions with cognitive and decision-making processes.
• **Bias-aware behavior modeling**: The experiments are explicitly designed to elicit and measure specific cognitive biases, loss aversion, base rate neglect, availability heuristic, confirmation bias, and sunk-cost fallacy, through carefully designed cyber 'triggers' embedded in the network environment. These triggers, grounded in cognitive science and behavioral economics, provide controlled manipulations to evaluate how biases influence tactical choices, persistence on suboptimal attack paths, and susceptibility to deception. The dataset thus supports the development, training, and validation of bias-sensitive analytics, classifiers, and adaptive defenses.
• **Benchmarking realism**: The experimental environment consists of an enterprise-like network topology with approximately 40 virtual machines per instance of the cyber range, populated with realistic services, user activity, and operational traffic. Each participant is provisioned with a standardized Kali “start box” for attack execution, along with realistic boundary conditions such as protected “no-strike” network segments. This fidelity allows the data set to serve as a benchmark for evaluating the ecological validity of cyber defense analytics and to compare performance between research teams, algorithms, and operational contexts.
• **Reusability and broad applicability**: Beyond raw telemetry, the dataset includes curated derivatives such as “clean logs,” labeled artifacts, and structured annotations of attacker–trigger encounters. These resources facilitate rapid onboarding into machine learning pipelines, reduce preprocessing burdens, and enable reproducibility studies. The data set is well suited for a range of research applications including training and evaluation of intrusion detection models and systems, studying attacker decision-making under stress of other cognitive states, development of cognitive vulnerability sensors, educational exercises such as cybersecurity training labs, and prototyping of proactive, adaptive, human-aware cyber defenses.

## 2 Background

The GAMBiT project was established to advance the empirical research on how cognitive biases influence adversary decision-making during realistic, operational-style cyber intrusions [4,5]. Although attacker behavior has been widely examined through theoretical models and simulated environments, there is a lack of high-fidelity, human-in-the-loop datasets that capture both detailed operational telemetry and the cognitive states underlying tactical choices.

GAMBiT aims to address this gap through a repeated-measures of red-team exercise format conducted in a fully instrumented, enterprise-grade cyber range. Within these exercises, controlled manipulations, referred to as cognitive-bias triggers, are embedded into realistic attack scenarios. These triggers are designed to induce measurable deviations from optimal or rational strategies and can be systematically attributed to specific biases such as loss aversion, base-rate neglect [6], confirmation and availability bias [7], and sunk-cost persistence [8]. The methodology integrates principles from cyber operations, experimental psychology, and human factors engineering, creating an empirical bridge between cognitive science theory and operational cybersecurity practice [9,10].

## 3 Data Description

The GAMBiT data set comprises three sequential human-subject red-team experiments: HSR1, HSR2, and HSR3 performed in an enterprise-grade cyber range designed for high ecological validity. Each experiment followed tightly controlled, repeatable procedures to enable rigorously measurement of attacker decision-making under varying cognitive-bias conditions. While sharing a common network architecture, standardized operational workflow, and synchronized multi-modal data capture framework, the experiments differed in trigger deployment, scenario refinements, and instrumentation depth. These controlled variations were deliberately introduced to distinguish scenario-driven effects from those attributable to cognitive biases, enabling both within-experiment and cross-experiment behavioral analyses. Table 1 summarizes the schedule, participant counts, and total data volume for each dataset release.

**Table 1:** Dataset scope by experiment: schedule, cohort size, and archive volume.

Experiment	Date (EST/EDT)	Participants	Data Size
GAMBiT Exp. 1 (HSR1)	2024-07-23 – 2024-09-14	19	722.8 GB
GAMBiT Exp. 2 (HSR2)	2024-11-09 – 2025-01-29	20	2.1TB
GAMBiT Exp. 3 (HSR3)	2025-02-01 – 2025-03-26	20	2.8TB

Experiment Summaries.

• **HSR1 (Baseline with Triggers)**: Established the foundational scenario and deployed a full suite of cognitive-bias triggers targeting loss aversion, base-rate neglect, availability bias, confirmation bias, and sunk cost persistence. Instrumentation emphasized host and network telemetry, supplemented with pre- and post-session psychometrics. Triggers were embedded along common attack paths to ensure encounter opportunities.
• **HSR2 (Control Condition)**: Removed all bias triggers to capture baseline attacker workflows and natural decision-making patterns. Instrumentation of the cyber range to collect human-subject behaviors was expanded to include hourly reasoning and affect surveys, enabling finer alignment between cognitive state and operational behavior. Network capture coverage was extended to additional vantage points, increasing the dataset’s scope relative to HSR1.
• **HSR3 (Refined Triggers)**: Reintroduced bias triggers in a more targeted and context-specific manner, informed by findings from HSR1 and HSR2. Refinements included timestamped encounter labels, co-occurring bias codes, and optimized placement to maximize encounter rates. Instrumentation upgrades featured enhanced keystroke partition by experimental time periods, expanded Suricata rule sets, and additional Expert Knowledge Model (EKM) sensors for near-real-time classification of “rational” versus “biased” actions.

Across all three phases, data capture was synchronized across modalities (network, host, and survey) with standardized file formats, metadata conventions, and consistent naming schemes to support automated parsing and robust cross-phase comparative analytics.

### 3.1 Top-level organization

**Fig. 1:**
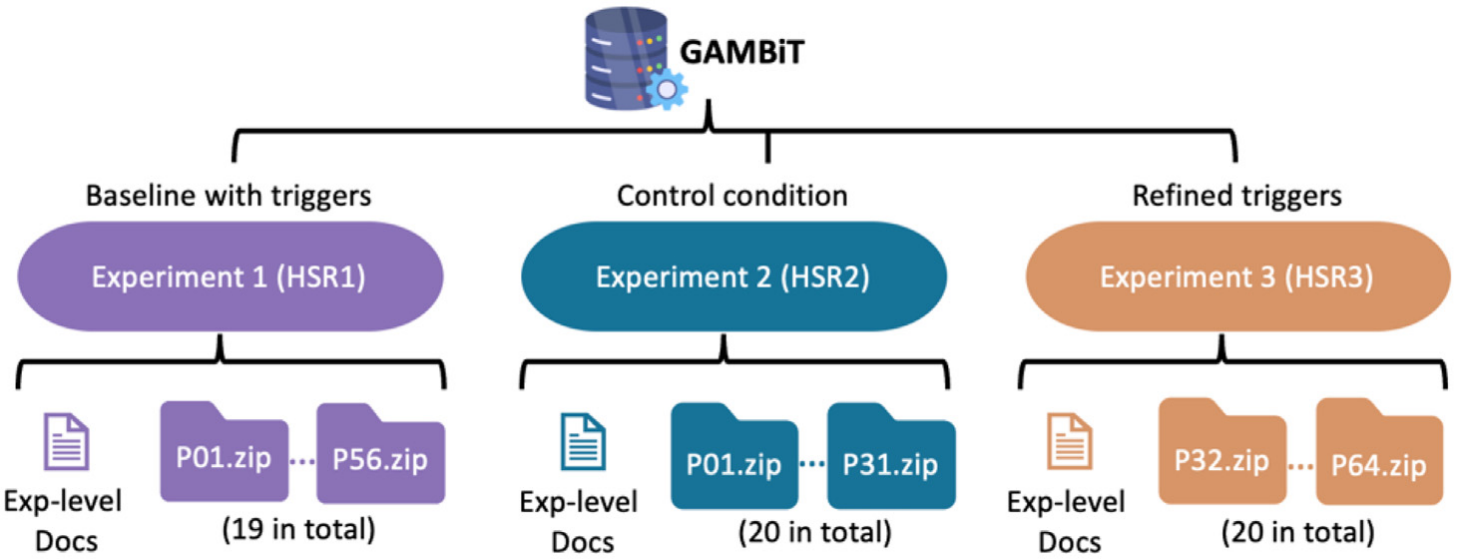
Top-level organization of the GAMBiT datasets.

At the archive root of each experiment (HSR1, HSR2, HSR3), there are: (i) a set of participant-indexed ZIP bundles, one per participant, and (ii) experiment-level documentation (README, schemas, checksums, notes), as illustrated in Fig. 1. Participant bundles are named with zero-padded numeric IDs, e.g., P01.zip, P02.zip, …, PXX.zip, preserving lexicographic order and simplifying programmatic access. There is also a cleaned-up spreadsheet (.xlsx) that contains the data on the screening, demographics, psychometrics, and Kali-command-to-MITRE-techniques for all experiment participants.

### 3.2 Per-participant contents (Modality Categories)

**Fig. 2:**
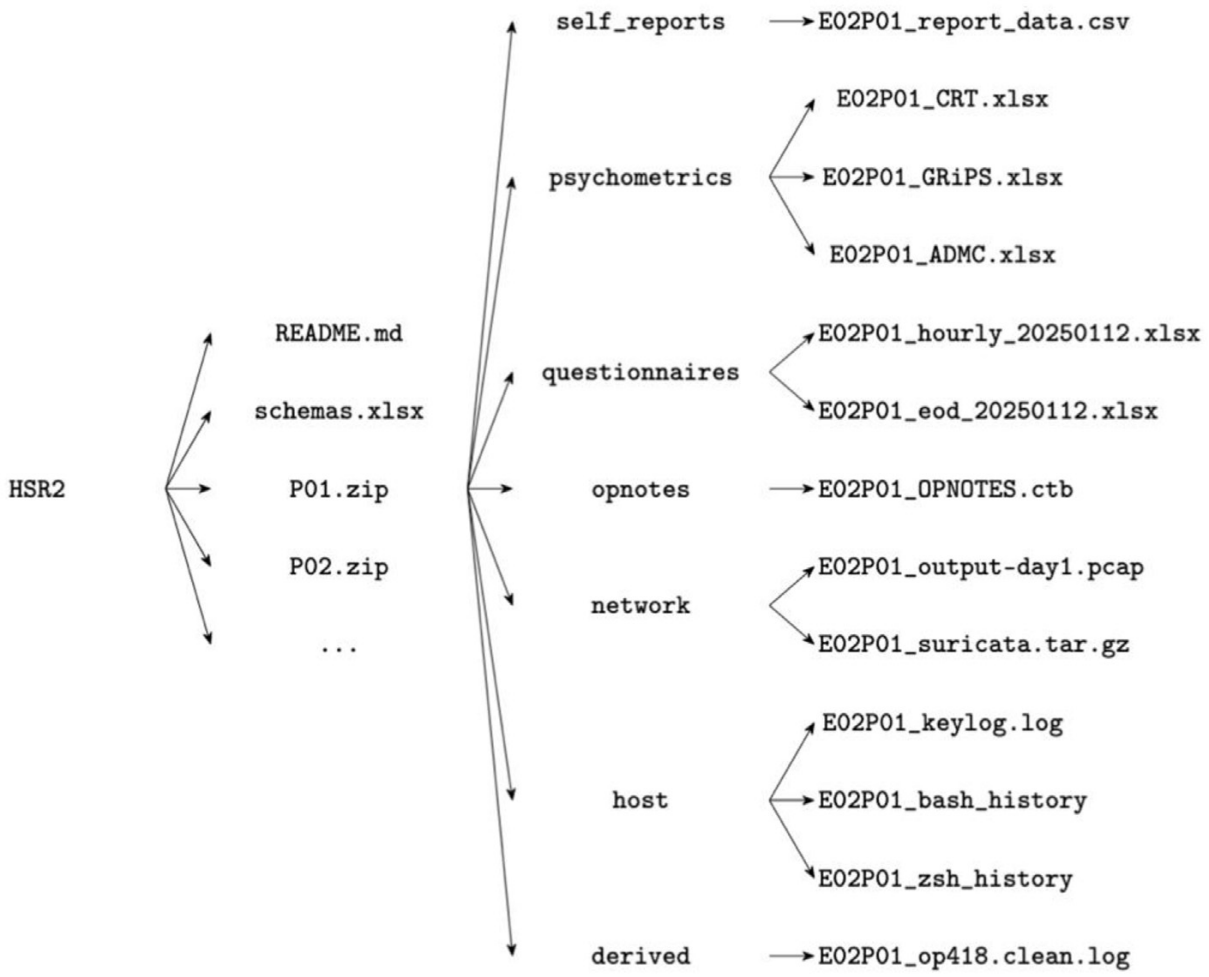
Directory layout - experiment-level files and participant-indexed bundles; each participant folder contains modality-specific and representative raw/derived files.

Within each participant ZIP, data can be organized by modality in a consistent hierarchy (Fig. 2):

• self-reports (or screening demographics): baseline screening & demographics in CSV/XLSX.
• psychometrics: CRT, BFI-2-XS, GRiPS, A-DMC item-level responses and scored sheets (CSV/XLSX).
• questionnaires: hourly and end-of-day reports (intended vs. applied ATT&CK, reasoning/affect in HSR2–HSR3), CSV/XLSX.
• opnotes: CherryTree operational notes (XML/CTB).
• network: PCAP captures and Suricata eve.json; may include summary tables (XLSX/CSV). Please note that large PCAP files are available only for Experiment 1; PCAP files for other experiments can be provided upon request (email: qz494@nyu.edu) due to their large sizes.
• host: keylogger streams, clipboard text, and timestamped .bash history/.zsh history.
• derived: curated “clean logs”, trigger-encounter annotations, and multi-modal alignment tables (CSV/XLSX).

### 3.3 Indexing and naming conventions

Participants are indexed numerically (P01, P02, . . .), and file names embed key descriptors to support automated fusion across modalities. A typical pattern is:

<ExpID><ParticipantID>_<Modality>-<YYYYMMDD-HHMMSS>.<ext>. For example, “E01P10_keylog.log” is the keylog file for experiment 1 participant 10; “E02P59_.ds-logs-apache.access-default-2025.03.19-000001.json.gz” is the Security Onion direct services logs associated with the active directory for experiment 2 participant 59 with timestamps. Timestamps are synchronized across modalities using the range time source (EST/EDT), enabling direct temporal alignment without additional preprocessing.

### 3.4 Directory diagram

Fig. 1 illustrates the top-level experiment → participant organization; participant→ modality hierarchy and representative files are presented in Fig. 2.

**Fig. 3:**
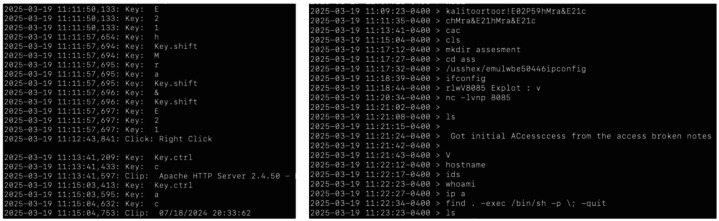
Excerpts of keylog.log (left) and op418.clean.log (right).

The release includes raw, processed, and derivative data across seven primary modalities, organized in a standardized directory structure:

• Self-reports: Baseline screening and demographic forms.
• Psychometrics: include the Cognitive Reflection Test (CRT), Big Five Inventory-2 Short/Extra-Short Forms (BFI-2-S / BFI-2-XS), General Risk Propensity Scale (GRiPS), and Adult Decision-Making Competence (A-DMC) subscales. Administered pre-scenario to characterize participant profiles.
• Questionnaires: Hourly and end-of-day forms capturing intended vs. applied MITRE ATT&CK techniques, operational reasoning, and affect/motivation measures (the latter added in HSR2–HSR3). These link subjective reasoning to objective behavioral traces.
• Operational Notes (OPNOTES): Participant-generated logs in CherryTree format, documenting perceived goals, network observations, and tactical decisions.
• Network data: Full packet captures (PCAP) from all subnets and Suricata intrusion detection alerts, aligned with the network clock and tagged per participant session. (Note that large PCAP files are available only for Experiment 1; PCAP files for other experiments can be provided upon request (email: qz494@nyu.edu) due to their large sizes.)
• Kali host data: Continuous keylogger streams and shell histories (.bash history, .zsh history), enabling reconstruction of exact command sequences with timing.
• Derivative products: Curated “clean logs” produced via running a post-processing script on Admin VM to improve readability and remove sensitive range management artifacts, suitable for direct use in analytics pipelines (Fig. 3).

The repository metadata describes the complete directory hierarchy, file naming conventions, and modality-specific schema definitions. Note that the screening, demographic, psychometrics, and Kali-command-to-MITRE-techniques alignment for all participants are organized into a cleaned-up spreadsheet (.xlsx) at the root of each experiment folder. For example, in the “Commands & MITRE Technique” tab, the headers are “Timestamp, Command, PID, MITRE Technique”, which maps the command executed by certain participant (PID) at certain time (Timestamp) to MITRE Technique (Table 2), aiming to make integration with existing tools easier. For further details on how the mapping is constructed, please refer to [11].

**Table 2:** Examples for commands and MITRE techniques mapping.

Timestamp	Command	PID	Technique
2025-02-12 10:53:51	nmap -p80 70.39.165.194/	E02P39	T1046: Network Service Discovery
2025-01-2310:13:41	proxychains nmap -sT -Pn -p88 172.16.7.103	E02P03	T1046: Network Service Discovery; T1090: Proxy
2025-01-15 17:00:17	source .zshrc	E02P20	T1059: Command and Scripting Interpreter
2024-12-04 11:05:16	cat id_rsa_it_admin	E02P17	T1083: File and Directory Discovery

## 4 Experimental Design, Materials and Methods

### 4.1 Cyber range architecture and experimental setup

The GAMBiT cyber range was provisioned on the SimSpace Cyber Force Platform and configured to emulate the operational environment of a mid-sized enterprise (SimSpace Business-Mini). The virtualized infrastructure comprised approximately 40 endpoints, including both server and workstation roles, running representative business services such as web hosting, file sharing, email, and directory services. Interconnection was achieved through virtual routers and switches configured with realistic internal subnetting and routing policies. To sustain an authentic background environment, synthetic user traffic, including web browsing, email exchanges, and file transfers, was continuously generated, camouflaging participant activity within normal operational patterns.

Each participant operated within a logically and physically isolated clone of a common baseline configuration. This isolation preserved experimental control, ensured repeatability, and prevented cross-contamination of telemetry across participants. The network replicated enterprise defense-in-depth architectures, including intrusion detection systems (IDS) and segmented security zones, to reinforce realism and operational complexity.

For more detailed setups, GAMBiT used Security Onion 2.4.20 on Oracle Linux 9, with Suricata, Zeek, Stenographer, and Strelka running in Docker as part of the standard Security Onion stack. Security Onion used the default community IDS ruleset, and all telemetry (Zeek logs, Suricata alerts/flows, endpoint logs, etc.) was forwarded into Elasticsearch.

The standardized starting point for all red-team operations was a Kali Linux virtual machine 10.10.0.5) provisioned with a curated toolkit for reconnaissance, exploitation, and post-exploitation. This uniform capability baseline allowed skill-level differences to emerge organically in operational traces.

To safeguard range management infrastructure and maintain exercise fidelity, several no-strike network segments were designated and enforced:

10.10.0.0/16 155.41.3.0/24 192.168.0.0/21 172.16.100.0/22 3.136.223.108.

Participants were instructed not to scan or interact with these segments, and violations were automatically detected and logged through firewall rules and monitoring agents.

**Fig. 4:**
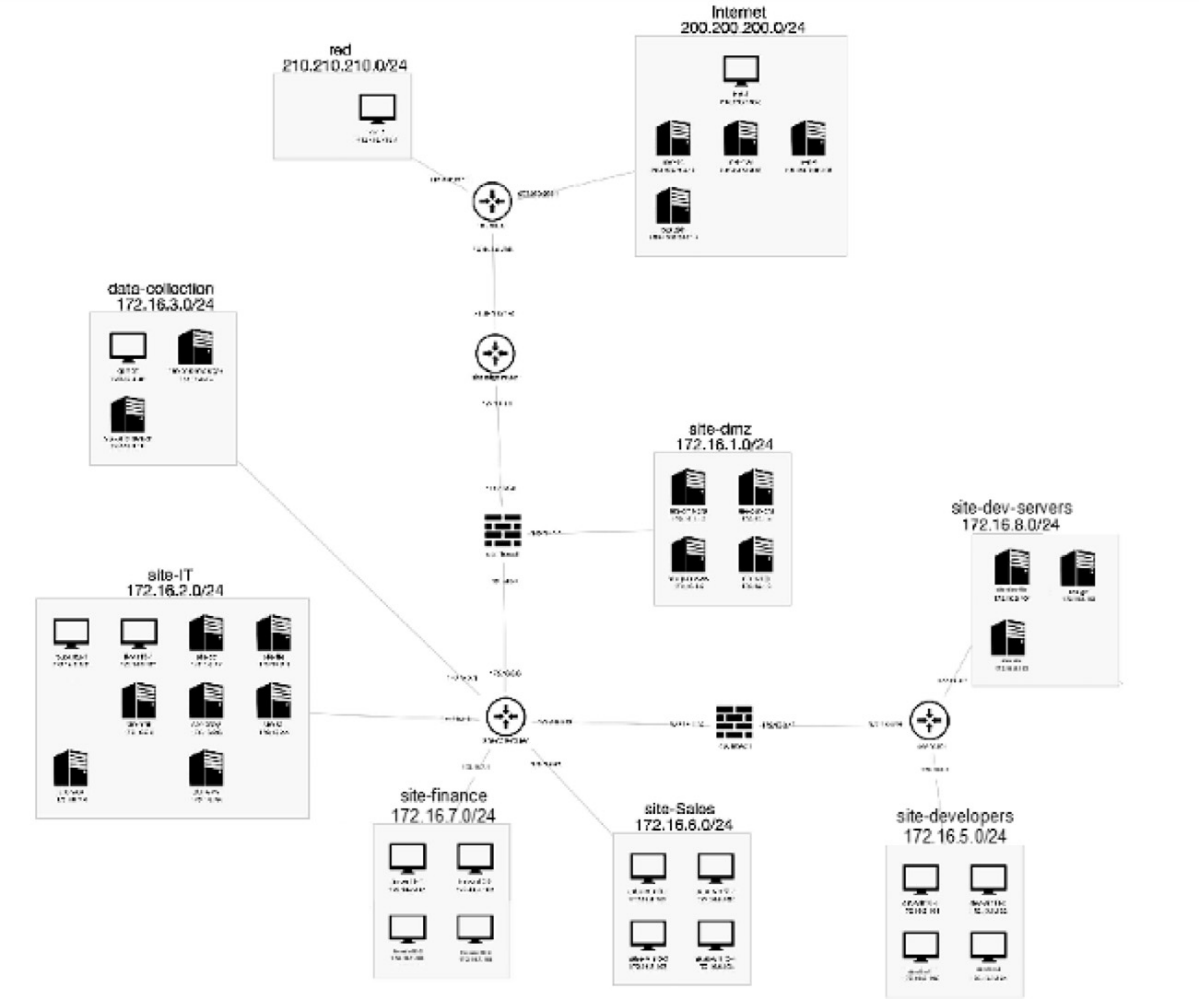
Representative GAMBiT cyber range topology showing core services, segmented zones, and participant start node.

The dataset release includes topology diagrams, IP allocation tables, and per-host service inventories. These resources detail OS distributions, active services, routing architectures, and simulated user account structures, enabling contextualization of captured network and host data (Fig. 4).

### 4.2 Bias triggers and labeling protocols

Bias triggers were embedded directly into the cyber range scenarios as controlled manipulations, jointly designed by cybersecurity SMEs and cognitive scientists to induce bias-prone decision-making under operationally realistic. Each trigger was mapped to a primary cognitive bias, linked to relevant MITRE ATT&CK tactics and techniques, and implemented in the network as a plausible file, credential, service, or configuration. The design process emphasized both technical plausibility and psychological efficacy, with iterative testing to ensure that each trigger could be encountered naturally along common attack paths. Representative examples include:

• **Loss Aversion**: A notes file referencing potentially valuable credentials that offered faster access but carried elevated detection risk, contrasted with a slower but safer exploitation.
• **Availability Heuristic**: Directories seeded with memorably named yet strategically irrelevant files, diverting attention from critical assets.
• **Base-Rate Neglect**: User accounts configured with misleading administrative naming conventions, encouraging unverified privilege assumptions.
• **Sunk Cost / Confirmation Bias**: Deceptive artifacts or aliased commands that incentivized persistence along unproductive or dead-end attack paths.

From HSR 1 to HSR 3, trigger placement was refined to increase encounter rates by positioning them in higher-traffic network locations and integrating supplementary logging sources for richer post-event. All triggers were labeled with a structured code indicating bias type, trigger class, and instance number (e.g., B.2.1.1 for a Base-Rate Neglect trigger of class 2, instance 1) to support rapid identification and consistent analysis.

In HSR2 and HSR3, the labeling framework was enhanced to include: (i) precise timestamps for each trigger encounter, (ii) standardized bias codes, and (iii) annotations from the Expert Knowledge Model (EKM) sensors indicating whether subsequent actions aligned with “rational” or “biased” decision. Table 3 provides illustrative examples on bias triggers. For example, B2.1.1 contained multiple “-adm” accounts are visible during discovery while sudo privileges are revoked in configuration files, creating a choice between verification commands and immediate escalation attempts. EKM sensors label verification as the rational path and immediate escalation as evidence of base-rate neglect. More detail on the design of triggers can be found in research paper [11].

**Table 3:** Examples for triggers, placements, bias types, associated MITRE techniques, and rational as well as biased decisions.

System	Trigger ID (Bias)	Trigger Type	Technique	Rational Decision	Biased Decision
site-www	A1.1 (Availability)	Mislabeled Application	Exploit Public-Facing Application (T1190)	Verify the version banner using tools before attempting exploitation.	Proceed with exploitation based solely on the version shown in the banner.
it-ubuntu-1	B2.1.1 (Base-Rate)	Mislabeled Accounts / Misconfigured Privilege	Account Discovery (TT1087) Permission Groups Discovery (T1069)	Attempt to switch user (su) into a single account to test privilege.	Attempt su repeatedly across multiple accounts without confirming actual privileges.
dev-win10-1	C9.2.1 (Confirmation)	False Information - Credentials	Unsecured Credentials (T1552)	Try the credentials in a few places and stop if unsuccessful.	Repeatedly try using the credentials without verifying them.

These refinements enabled fine-grained statistical and modeling analyses of how specific cognitive biases influenced adversary decision-making, revealing measurable impacts on attacker progress, resource expenditure, and operational detectability in complex, time-constrained operations.

### 4.3 Scenario and objectives

Each experiment placed participants in a realistic, enterprise-scale cyber range simulating a mid-sized organization with multiple subnets, operational services, and user traffic. Participants were given initial access from a standardized Kali Linux ``start box'' and a briefing outlining broad operational objectives: infiltrate the network, expand access, map services, identify high-value assets, and exfiltrate sensitive files. Note that the participants were not informed of any exact cyber deception or security measures being deployed in the cyber range and they were not aware of which experimental groups (with or without triggers) they were assigned.

The scenario only assigned hacking objectives without prescribing any procedures and actions, allowing participants to self-direct their tactics, techniques, and procedures (TTPs) over the course of a multi-stage, multi-hour engagement. Across the experiments, objectives were periodically updated every four hours to simulate evolving mission priorities (e.g., collect system administrator details, enumerate developer network, or acquire finance records).

Participants recorded their thought processes, decision points, and operational steps through:

• Hourly reports – detailing intended and executed MITRE ATT&CK techniques, as well as responses to targeted reasoning and affect questions (in Exp. 2–3).
• Structured operational notes (CherryTree format) – providing narrative context, network mapping results, and intermediate findings.
• End-of-day summaries – reflecting on successes, challenges, engagement level, and perceived adaptation.

This design aimed to enable direct linkage of subjective reasoning and affect with objective behavioral telemetry, facilitating both cognitive and operational analysis.

### 4.4 Environment and Instrumentation

Experiments were conducted using the SimSpace Cyber Force Platform, which provisioned identical, isolated ranges for each participant. The environment was preconfigured with ∼40 virtual hosts spanning finance, sales, developer, and DMZ segments, with realistic business services and synthetic user traffic. Instrumentation spanned three primary layers:

• Network-level capture: Full packet capture (PCAP) at range egress points; Suricata IDS rulesets tuned to detect suspicious or policy-violating traffic, generating time-stamped alerts for correlation with host and survey data.
• Host-level capture (Kali start box): Continuous keylogging, including keystrokes and clipboard activity, to capture reconnaissance, exploitation, and post-exploitation commands. Timestamped Bash and Zsh command histories to support command sequence reconstruction.
• Human-subjects instruments: Pre-scenario psychometric batteries — Cognitive Reflection Test (CRT), Big Five Inventory-2 Extra-Short Form (BFI-2-XS), General Risk Propensity Scale (GRiPS), and Adult Decision-Making Competence (A-DMC) subscales. Hourly and end-of-day surveys capturing tactics used, reasoning, affective state, and trigger recognition (Exp. 2–3 added Likert-scale reasoning/affect items).

All telemetry streams were clock-synchronized to enable cross-modal correlation.

### 4.5 Derived artifacts

In addition to raw captures, the dataset includes derivative products to support rapid analysis:

• Curated “clean logs” – keylogger streams resulted from running a post-processing script on Admin VM to remove extraneous or repeated keystrokes while preserving operational context and temporal fidelity.
• Multi-modal alignment files – per-participant, per-session aggregates linking network events, host commands, IDS alerts, and survey responses into a unified timeline.
• Trigger encounter annotations – labeling instances where participants interacted with bias-targeted artifacts, noting associated MITRE ATT&CK techniques and Expert Knowledge Model (EKM) bias sensor outputs.

### 4.6 Hypotheses and bias constructs

The GAMBiT experimental framework was designed to evaluate two central hypotheses. First, attacker proficiency level, operationalized as division assignment (open vs. expert), predicts the rate of progress along a predefined “ideal” attack path, which represents the most efficient sequence of steps leading to scenario objectives. Second, the deliberate introduction of cognitive-bias triggers into the operational environment produces measurable deviations in attacker decision-making, resource allocation, and tactical sequencing. Bias constructs were instantiated in scenario design through specific, controlled stimuli intended to elicit well-documented cognitive heuristics and systematic errors. These included:

• Loss aversion: The tendency to overvalue the preservation of existing gains, such as persisting with a risky shortcut (e.g., planted credentials) rather than transitioning to a slower but more reliable exploitation method.
• Base-rate neglect: Disregarding prior probability distributions, for example assuming that accounts labeled with “-adm” automatically possess elevated privileges without verification.
• Availability heuristic: Overweighting salient or recent information, such as prioritizing files with conspicuous names regardless of actual content relevance.
• Confirmation bias: Selectively seeking evidence that reinforces an existing working hypothesis, for instance repeatedly attempting to use invalid SSH keys while rationalizing authentication failures.
• Sunk cost fallacy: Persisting with ineffective tactics due to previous investment of time or effort, such as continuously targeting a protected file of minimal strategic value.

By combining these targeted bias-inducing manipulations with high-fidelity operational data and synchronized human-subject measurements, GAMBiT enables rigorous empirical analysis of attacker cognition, decision-making under uncertainty, and susceptibility to defensive deception. This design supports both statistical inference and computational modeling of bias dynamics in adversarial contexts, offering an empirical basis for bias-aware defensive strategies.

### 4.7 Demographic summary

Sixty-one participants in total completed the three experiments. The age of the participants ranged between 19 and 43 years (M = 26.8, SD = 5.4). The participants were predominantly male (59 males, 96.7%; 2 females, 3.3%). Most participants reported their country of origin to be India (42.6%) or the United States (21.3%), with the remaining participants (36.1%) coming from Lebanon, Malaysia, Indonesia, Singapore, Israel, Egypt, Hong Kong, Syria, Kazakhstan, Brazil, Portugal, Taiwan, Saudi Arabia, Vietnam, and Pakistan. Approximately half reported their native language to be English (47.5%). In terms of employment, 57.4% were employed full-time, 18.0% employed part-time, 23.0% students, and 1.6% unemployed. More details are provided in Table 4.

**Table 4:** Participant demographics and hacking experience (N = 61).

Variable	% or M (SD)
Age (years)	M = 26.8 (SD = 5.4), range 19–43
Gender	Male (n = 59; 96.7%), Female (n = 2; 3.3%)
Country of origin	India (n = 26; 42.6%), USA (n = 13; 21.3%), other countries (n = 22; 36.1%)
Native English speaker	Yes (n = 29; 47.5%), No (n = 32; 52.5%)
Employment status	Full time (n = 35; 57.4%), Part time (n = 11; 18.0%), Students (n = 14; 23.0%), Unemployed (n = 1; 1.6%)
Hacking as the source of income	Primary (n = 28; 45.9%), Secondary (n = 17; 27.9%), None (n = 14; 23.0%), Other (n = 2; 3.3%)
Years of hacking experience (professionally)	<1 year (n = 9; 14.8%), 1–3 years (n = 30; 49.2%), 4–6 years (n = 11; 18.0%), 7–10 years (n = 4; 6.6%), None (n = 7; 11.5%)
Years of hacking experience (personally)	1–3 years (n = 29; 47.5%), 4–6 years (n = 20; 32.8%), 7–10 (n = 9; 14.8%), ≥10 years (n = 3; 4.9%)
Cybersecurity certification held	Yes (n = 49; 80.3%), No (n = 12; 19.7%)
Hackathon/CTF participation	1–5 events (n = 26; 42.6%), 6–10 events (n = 14; 23.0%), 11–15 events (n = 5; 8.2%), ≥16 events (n = 9; 14.8%), None (n = 7; 11.5%)

Many participants reported that hacking was a professional activity with 45.9% reported hacking as their primary source of income, 27.9% as a secondary source, and 23.0% reported no hacking-related income. Participants had substantial experience in professional roles requiring hacking skills, with 14.8% less than 1 year, 49.2% between 1 and 3 years, 18.0% between 4 and 6 years, and 6.6% between 7 and 10 years; 11.5% without any professional roles requiring hacking skills. For participants who were hobbyist hackers, 47.5% had engaged in hacking between 1 and 3 years, 32.8% between 4 and 6 years, 14.8% between 7 and 10 years, and 4.9% with more than 10 years. Most participants (80.3%) reported holding at least one cybersecurity certification. Nearly all had prior experience with hackathons or capture-the-flag competitions throughout their lifetimes, with 42.6% reporting participation between 1 and 5 events, 23.0% between 6 and 10, 8.2% between 11 and 15, and 14.8% in 16 or more, while 11.5% reported no competition experience.

### 4.8 Recruitment/screening summary

**Fig. 5:**
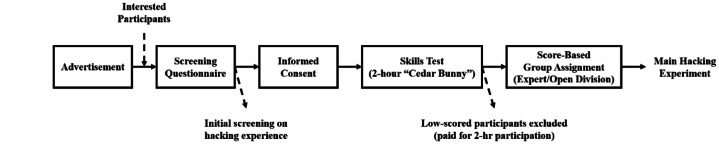
Overview of recruitment procedures.

Recruitment involved the following five steps (Fig. 5):

1. Advertisement: To appeal to hacker communities, the experiment was branded “Operation 418” and framed as a themed hacking competition. Participants were offered hourly compensation for their time ($15/hour) and additional prize money if they were the best performer in their assigned participant group (i.e., $300 and $750 in Open and Expert divisions, respectively). To reach both experienced and amateur hackers, the research team reached out to cyber competition administrators, leaders of cyber workgroups, and professional contacts. A recruitment flyer was also disseminated on professional social media platforms (e.g., LinkedIn).
2. Screening: To ensure that participants could complete a substantial portion of the hacking exercise and were reasonably representative of the target population, applicants who contacted the experimenters first completed a screening questionnaire to determine eligibility based on inclusion criteria (Table 5).

**Table 5:** Inclusion criteria for the screening questionnaire.

**Eligibility**	**> 18 years old** **> 1 years of hacking experience** in either hobbies or professional **> 10 hours/week in hacking** in recent 1 year
**Basic skills in environment**	**Expressed familiarity to Kali Linux ≥ 3** (Not at all = 1, Extremely Familiar = 5)
**Basic proficiency in core skills**	**Self-rated skill level ≥ 2** for all skills (Don’t know = 0, Novice = 1, Expert = 5) - Information Gathering - Locating Public Exploits - Tunneling and Pivoting - Using Command and Control Framework
**Basic proficiency in other skills**	**Self-rated skill level ≥ 1** for all skills (Don’t know = 0, Novice = 1, Expert = 5) - Active Directory Enumeration & Attacks - Buffer Overflows - Client-Side Attacks - Enumerating Remote Services - Password Attacks & Cracking - Port Scanning & Redirection - Privilege Escalation - Scripting - SQL Injection Attacks - Target Reconnaissance & Vulnerability Scanning - Web Application Attacks
**Willingness to participate**	Respond ‘**Yes**’ to the willingness to participate in two-day experiment (∼8 hrs a day)
3. Informed consent: Individuals who passed the initial screening were asked to provide informed consent form that included description of the experiment.
4. Cybersecurity Skills Test: Upon providing consent, participants were instructed to complete the “Cedar Bunny” cybersecurity skills assessment in SimSpace as a second-stage eligibility examination. This assessment combined multiple-choice items with Capture-the-Flag (CTF) tasks of network enumeration, vulnerability scanning, exploitation, and privilege escalation. Cedar Bunny scores range from 0 to 1200. Participants attaining a score 420 or above were invited for participation in the two-day hacking experiment. Those who did not pass the threshold were compensated at the hourly compensation rate.
5. Experimental Group Assignment: Participants scoring above 800 points were assigned to the Expert division, whereas those scoring between 420 and 800 points were assigned to the Open division.

### 4.9 Usage notes

The GAMBiT dataset is distributed as three experiment-specific archives (HSR1, HSR2, HSR3), each hosted on IEEE Dataport with persistent DOI links provided in the Specifications Table. This segmentation allows users to selectively download only the experiment relevant to their analysis or to work across all three for comparative studies.

### 4.10 Recommended tools

Due to its multi-modal nature, the dataset aims to be approached with a variety of analytical toolchains, depending on the user’s domain focus and methodological expertise. The following recommendations are not exhaustive but represent common starting points for working with GAMBiT data:

• Network captures (PCAP) and IDS alerts: Tools such as *tcpdump* can be used for targeted packet filtering and extraction, while *tshark* or Wireshark provide interactive packet inspection with protocol-level decoding. Suricata’s *eve.json* output can be parsed using Python or Logstash pipelines for signature-based intrusion event analysis and correlation with other modalities.
• Host-level logs (keystrokes, shell histories): Standard text-processing utilities like *grep, ripgrep*, or *awk* are useful for rapid keyword searches. For structured log parsing and temporal analysis, JSON processors like *jq* and data analysis libraries in Python (*pandas*) or R (*dplyr, tidyverse*) enable session segmentation, command sequence reconstruction, and feature extraction for statistical or machine learning models.
• Survey and psychometric data: The structured CSV and JSON formats are compatible with statistical packages in Python (*pandas, numpy, scipy, statsmodels*) and R (*psych, tidyverse*). These can be used to score psychometric scales, compute internal consistency measures (e.g., Cronbach’s alpha), perform inferential tests, or integrate self-report measures with operational telemetry for mixed-methods analysis.

To streamline multi-modal analysis, all data streams have been normalized to a consistent timestamp format and synchronized to the range’s NTP service. This enables straightforward cross-referencing of events between network, host, and self-report modalities without the need for substantial temporal alignment preprocessing. Where possible, file naming conventions embed participant identifiers, modality codes, and session timestamps, further reducing the overhead in linking disparate data sources.

### 4.11 Use cases

There are several use cases for this dataset, spanning cybersecurity research, machine learning, human factors, education, and strategic policy work. The combination of multi-modal telemetry, human factors data, and controlled yet realistic enterprise-like scenarios enables both methodological development and empirical validation.

1. Cybersecurity Research and Analytics: The dataset supports investigations into attacker behavior modeling, adversarial decision-making, and cognitive bias analysis. By linking operational telemetry with survey-based self-reports, researchers can study how decision-making evolves under bias-triggering conditions, examine the relationship between attacker skill indicators and operational outcomes, and compare the effectiveness of heuristic versus machine learning and large language models approaches in detecting behavioral signatures of bias [12]. These capabilities make the dataset suited for work in adversarial reasoning, deception detection, and behavior-based intrusion detection [13].

2. Machine Learning and AI Benchmarking: The curated “clean logs” and labeled annotations enable reproducible benchmarks for predictive models, such as sequence classification to anticipate the next command or tactic, bias detection models that integrate host, network and psychometric features, and cross-modal fusion architectures that combine text, time series and network data. Because the scenarios capture realistic and varied attacker workflows, they are ideal for assessing the robustness and generalizability of machine learning pipelines.

3. Human Factors and Cognitive Security Studies

The inclusion of psychometric and affective measures offers a rare opportunity to link cognitive traits and decision-making processes to operational performance. Researchers can explore how individual differences, such as cognitive reflection or risk perception [14], influence cyber operations, compare self-reported reasoning to observed actions, and assess correlations between bias susceptibility and task efficiency or success. This supports studies in human-in-the-loop security, cognitive load modeling, and attacker persona profiling [15].

4. Education and Training

Because the environment is realistic yet fully controlled, the dataset serves as a resource for cybersecurity curriculum development, where students can analyze authentic adversary behavior without direct system access. It also supports red team/blue team training exercises and digital forensics practice, using PCAP and host telemetry for event reconstruction. Replayable scenarios and annotated datasets allow learners to experiment with diverse analytical approaches.

#### 4.11.1 Policy and strategy development

Beyond technical and educational applications, the dataset informs policy-oriented research by providing empirical evidence for bias-aware defensive measures, enabling evaluation of training programs designed to reduce decision-making errors, and supporting wargaming scenarios that incorporate realistic attacker cognition models [4,16]. Its structure, annotations, and multi-modal scope make it valuable to both practitioners and decision-makers seeking data-driven insights.

### 4.12 Related datasets

Several publicly available datasets capture human behavior and decision-making in cybersecurity contexts, with explicit or implicit connections to cognitive bias. These resources provide valuable baselines and complementary data for studying the mechanisms underlying biased judgment, susceptibility to manipulation, and operational decision-making under uncertainty.

The PsyScam dataset [17] is a curated collection of online scam communications, sourced from multiple public reporting platforms, and annotated according to established psychological and cognitive influence techniques. It supports supervised learning tasks such as tactic classification, scam type identification, and automated generation of realistic deceptive content. By grounding annotations in cognitive science theory, PsyScam enables systematic study of persuasion mechanisms relevant to social engineering and phishing.

The Cry Wolf dataset [18] investigates the phenomenon of alert fatigue in cybersecurity operations, focusing on analyst responses to intrusion detection system alerts, particularly “impossible travel” events. It contains both alert metadata and analyst classification outcomes (true positive vs. false positive), making it suitable for analyzing decision-making degradation in noisy environments. While not explicitly bias-annotated, the dataset aligns with research on cognitive overload and trust calibration in human-machine teaming.

Developed under the IARPA ReSCIND program, the PsyCCDEF dataset [19] embeds classic cognitive bias elicitation tasks within ecologically valid cyber problem-solving scenarios. Participant responses, decision times, and accuracy are recorded, demonstrating that 81% of observed biases from the traditional literature also manifest in cyber-relevant settings, often with measurable performance costs. This dataset offers a rare opportunity to examine bias persistence in domain-specific operational contexts.

While the above datasets each contribute valuable insights, our datasets differ in several key aspects. First, it combines *multi-modal raw telemetry* (e.g., network packet captures, host-level logs, keystroke data) with *fine-grained psychometric assessments* administered longitudinally across experimental sessions, enabling cross-temporal modeling of cognitive bias dynamics. Second, unlike the CTF and Cry Wolf datasets, which focus on narrowly scoped operational tasks, HSR encompasses a *broad task ecology* including self-report surveys, psychometrics, scripted cyber exercises, and naturalistic behavioral interactions. Third, compared to PsyScam, which contains only attacker-generated content, HSR records both stimuli and participant behavioral responses, making it suitable for end-to-end modeling of bias influence and mitigation. Finally, PsyCCDEF places bias measurement within cyber scenarios but does not include rich raw data streams for replay or forensic analysis. The inclusion of such streams in our datasets facilitates multi-level analysis spanning cognitive, behavioral, and system-interaction layers.

## Limitations

No-strike segments constrain some reconnaissance behaviors; range artifacts (triggers) may shape action sequences; keylogging and shell histories reflect the Kali jump host (other hosts instrumented via network vantage). Survey compliance/timing may vary by participant.

## Ethics Statement

Data were collected as human-subjects research (HSR) with informed procedures appropriate to the program and context. Releases include operational notes and survey responses from consenting participants, with data curated for research access via controlled repositories. Users should adhere to the repository access terms and any downstream IRB requirements. The study protocol was reviewed and approved by the Institutional Review Board (IRB) at Virginia Tech (IRB# 23-565). All participants provided informed consent prior to data collection, acknowledging de-identified data would be shared for scientific purposes.

## CRediT Author Statement

All authors contributed to the conceptualization, development of the methodology, and formal analysis of the dataset.

## Data Availability

IEEE DataPortGuarding Against Malicious Biased Threats (GAMBiT) Experiment 2 (Original data)
